# Utility of a simplified [^18^F] sodium fluoride PET imaging method to quantify bone metabolic flux for a wide range of clinical applications

**DOI:** 10.3389/fendo.2023.1236881

**Published:** 2023-09-13

**Authors:** Tanuj Puri, Michelle L. Frost, Amelia E. B. Moore, Ananya Choudhury, Sobhan Vinjamuri, Abhishek Mahajan, Claire Fynbo, Marie Vrist, Jørn Theil, Kalevi Kairemo, James Wong, Habib Zaidi, Mona-Elisabeth Revheim, Thomas J. Werner, Abass Alavi, Gary J. R. Cook, Glen M. Blake

**Affiliations:** ^1^ Faculty of Biology, Medicine and Health, School of Medical Sciences, Division of Cancer Sciences, The University of Manchester, The Christie NHS Foundation Trust, Manchester, United Kingdom; ^2^ Department of Biomedical Engineering, School of Biomedical Engineering and Imaging Sciences, King’s College London, St. Thomas’ Hospital, London, United Kingdom; ^3^ Institute of Cancer Research Clinical Trials & Statistics Unit (ICR-CTSU), The Institute of Cancer Research, Sutton, United Kingdom; ^4^ Department of Cancer Imaging, and King’s College London and Guy’s and St Thomas’ PET Centre, School of Biomedical Engineering and Imaging Sciences, King’s College London, St. Thomas’ Hospital, London, United Kingdom; ^5^ Nuclear Medicine Department, Royal Liverpool and Broadgreen University Hospitals NHS Trust, Liverpool, United Kingdom; ^6^ The Clatterbridge Cancer Centre NHS Foundation Trust, University of Liverpool, Liverpool, United Kingdom; ^7^ Clinic of Nuclear Medicine, Gødstrup Hospital, Herning, Denmark; ^8^ University Clinic in Nephrology and Hypertension, Gødstrup Hospital, Herning, Denmark; ^9^ Department of Clinical Medicine, Aarhus University, Aarhus, Denmark; ^10^ Department of Nuclear Medicine, The University of Texas MD Anderson Cancer Center, Houston, TX, United States; ^11^ Department of Anaesthesia, St Bartholomew’s Hospital, Barts Health NHS Trust, London, United Kingdom; ^12^ Geneva University Hospital, Division of Nuclear Medicine and Molecular Imaging, Geneva, Switzerland; ^13^ The Intervention Centre, Oslo University Hospital, Norway Institute of Clinical Medicine, University of Oslo, Oslo, Norway; ^14^ Department of Radiology, Hospital of the University of Pennsylvania, Philadelphia, PA, United States

**Keywords:** quantitative measurement of bone, bone metabolic flux, clinical applications, future developments, [^18^F]NaF, K_i_, PET-CT, [^18^F] sodium fluoride

## Abstract

We review the rationale, methodology, and clinical utility of quantitative [^18^F] sodium fluoride ([^18^F]NaF) positron emission tomography-computed tomography (PET-CT) imaging to measure bone metabolic flux (K_i_, also known as bone plasma clearance), a measurement indicative of the local rate of bone formation at the chosen region of interest. We review the bone remodelling cycle and explain what aspects of bone remodelling are addressed by [^18^F]NaF PET-CT. We explain how the technique works, what measurements are involved, and what makes [^18^F]NaF PET-CT a useful tool for the study of bone remodelling. We discuss how these measurements can be simplified without loss of accuracy to make the technique more accessible. Finally, we briefly review some key clinical applications and discuss the potential for future developments. We hope that the simplified method described here will assist in promoting the wider use of the technique.

## 1 Introduction

Recent years have seen a resurgence of interest in the use of [^18^F] sodium fluoride ([^18^F]NaF) positron emission tomography-computed tomography (PET-CT) imaging for the investigation of skeletal diseases (1). [^18^F]NaF PET-CT offers several advantages over traditional gamma-camera imaging, including higher spatial resolution and improved image contrast due to the superior imaging properties of [^18^F]NaF (2). Additionally, due to the absence of protein binding and the rapid clearance from soft tissue, image acquisition can start as early as 1 hour after tracer injection. Despite these advantages, traditional bone imaging agents such as technetium-99m-hydroxy diphosphonate ([^99m^Tc]-HDP) and technetium-99m-labelled diphosphonate ([^99m^Tc]-MDP) are still often employed in clinical settings. This has been attributed to the limited availability of PET scanners and [^18^F]NaF tracer and the higher costs associated with PET scans compared with gamma-camera imaging. However, the landscape is evolving rapidly, with an increasing preference for [^18^F]NaF PET. The availability of [^18^F]-labelled radionuclides in Nuclear Medicine facilities is on the rise worldwide due to the growing demand for [^18^F] fluorodeoxyglucose ([^18^F]FDG), thereby reducing the cost per PET scan. In addition, the global market shortage of technetium-99m in 2010 raised significant concerns regarding its future use, thereby increasing the cost of gamma camera scans. Furthermore, notable advancements in PET scan technology, such as improved time-of-flight (ToF) corrections, depth-encoded fully digital detectors with silicon photomultiplier (SiPM) readouts, and total-body PET systems with an extended cylindrical field-of-view, will substantially enhance photon count sensitivity compared to older scanners. Consequently, these advancements enable high-quality whole-body PET image acquisition to be performed with faster scanning times and lower radiation doses.

Unlike the more familiar technique of [^18^F]FDG, which images malignant disease, quantitative [^18^F]NaF PET-CT scans measure bone metabolic flux (K_i_, alternatively known as regional bone plasma clearance), a measure that correlates with the rate of bone formation at the measurement site (3, 4). The conventional protocol for performing the studies has involved an hour-long dynamic PET scan at a single bed position combined with arterial blood sampling to measure the arterial input function (AIF) (5). We have previously described a simplified imaging protocol based on the combination of a short (3 or 4-minute) static PET-CT scan acquired 1-hour after tracer injection with 2 or 3 venous blood samples at 30 to 60 minutes after injection to determine the terminal exponential of the arterial input function (AIF) (6). The terminal exponential is added to a population average residual function representing the peak of the bolus injection and the early fast exponential components to approximate the full 0 to 60 min AIF (7). An important advantage of the technique is that a series of short static scans can be acquired at several bed positions, enabling K_i_ values to be measured at multiple sites with only a single injection of tracer. A major aim of the method is to achieve the best possible accuracy and precision (i.e., the lowest reproducibility errors) in order to minimise the number of participants required in research studies at the desired level of significance.

In this article, we first review the bone remodelling cycle and explain what aspects of the cycle are measured by quantitative radionuclide imaging methods such as [^18^F]NaF PET-CT. We explain how the technique works, what measurements are involved, and what makes [^18^F]NaF PET-CT a valuable tool for the study of bone metabolism. We discuss what types of measurements are required and how these measurements can be simplified without loss of accuracy to make the technique more accessible. Finally, we review some existing clinical applications and the potential for future developments. We hope that by shortening the technique, providing access to a data collection sheet to record the basic information necessary for the calculation of K_i_ values, and an Excel spreadsheet to simplify them (6), this review will assist the wider use of quantitative [^18^F]NaF PET-CT in the community.

## 2 What is bone remodelling?

**Figure 1:**
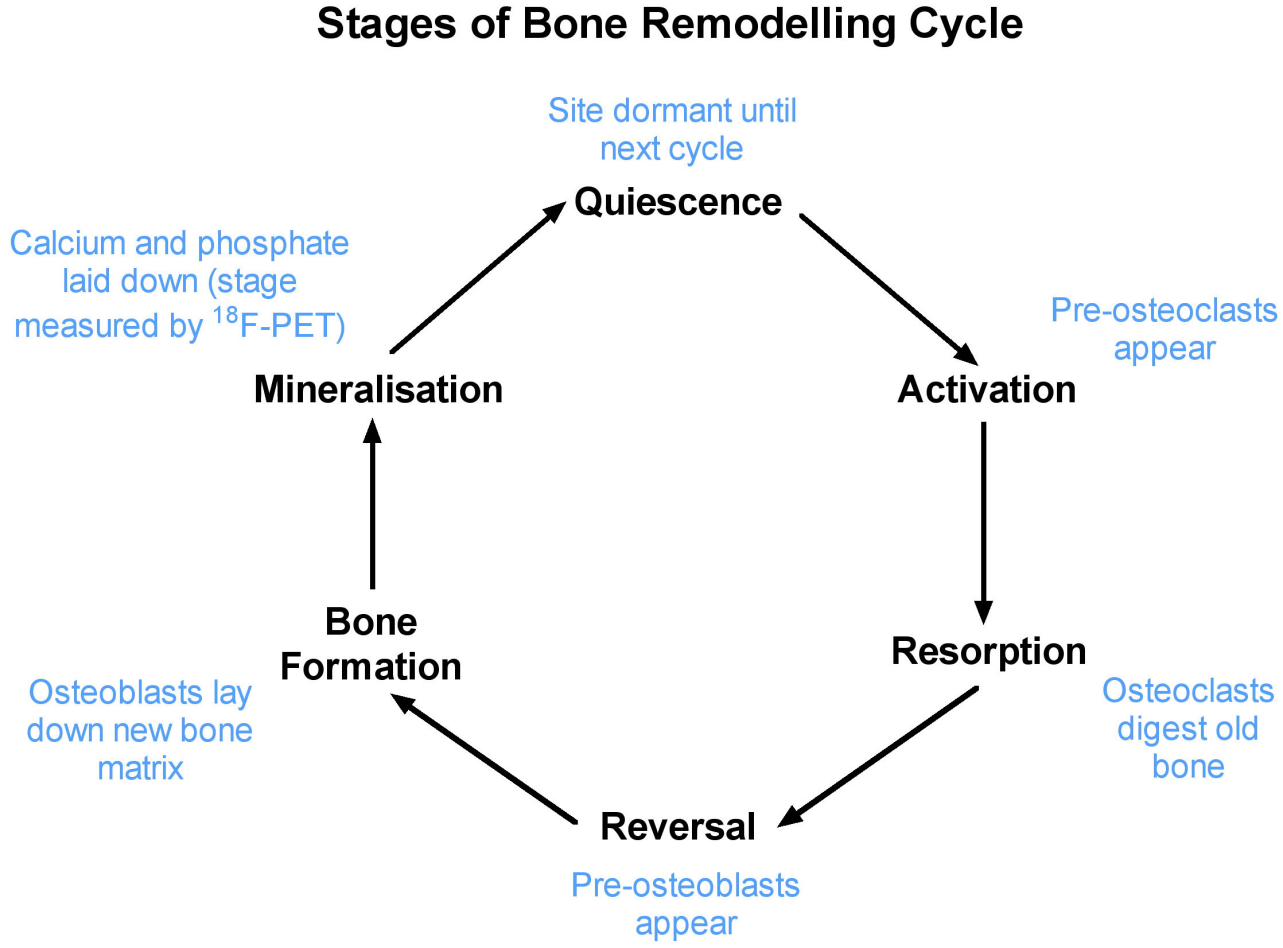
The six stages involved in bone remodelling process. Bone seeking radionuclides are incorporated into bone during the mineralisation stage.

Bone is a living tissue that requires continuous repair and renewal throughout life, a process known as bone remodelling. Bone remodelling is an active metabolic process that involves a dynamic interplay between osteocytes, osteoclasts, and osteoblasts that removes old bone tissue and replaces it with new bone, such that over a period of 5 to 10 years, the entire skeleton is rebuilt (8). Bone remodelling is a cyclic process (**Figure 1**). At the start of the cycle, the bone site is in a quiescent phase until osteocytes responding to local mechanical and chemical stimuli initiate the activation stage, at which point cells lining the bone surface transform into osteoclasts. Subsequently, during the resorption phase, the osteoclasts digest the old bone, followed by a reversal phase mediated by mononuclear cells, leading to the appearance of osteoblasts on the surface of the bone resorption pit. The subsequent bone formation stage involves the deposition of new bone matrix by osteoblasts. This is followed by the mineralisation stage, during which calcium, phosphate, and hydroxyl ions are incorporated into the bone matrix to form hydroxyapatite [Ca_10_(PO_4_)_6_(OH)_2_], at the completion of which the cycle returns to the quiescent state.

**Figure 2:**
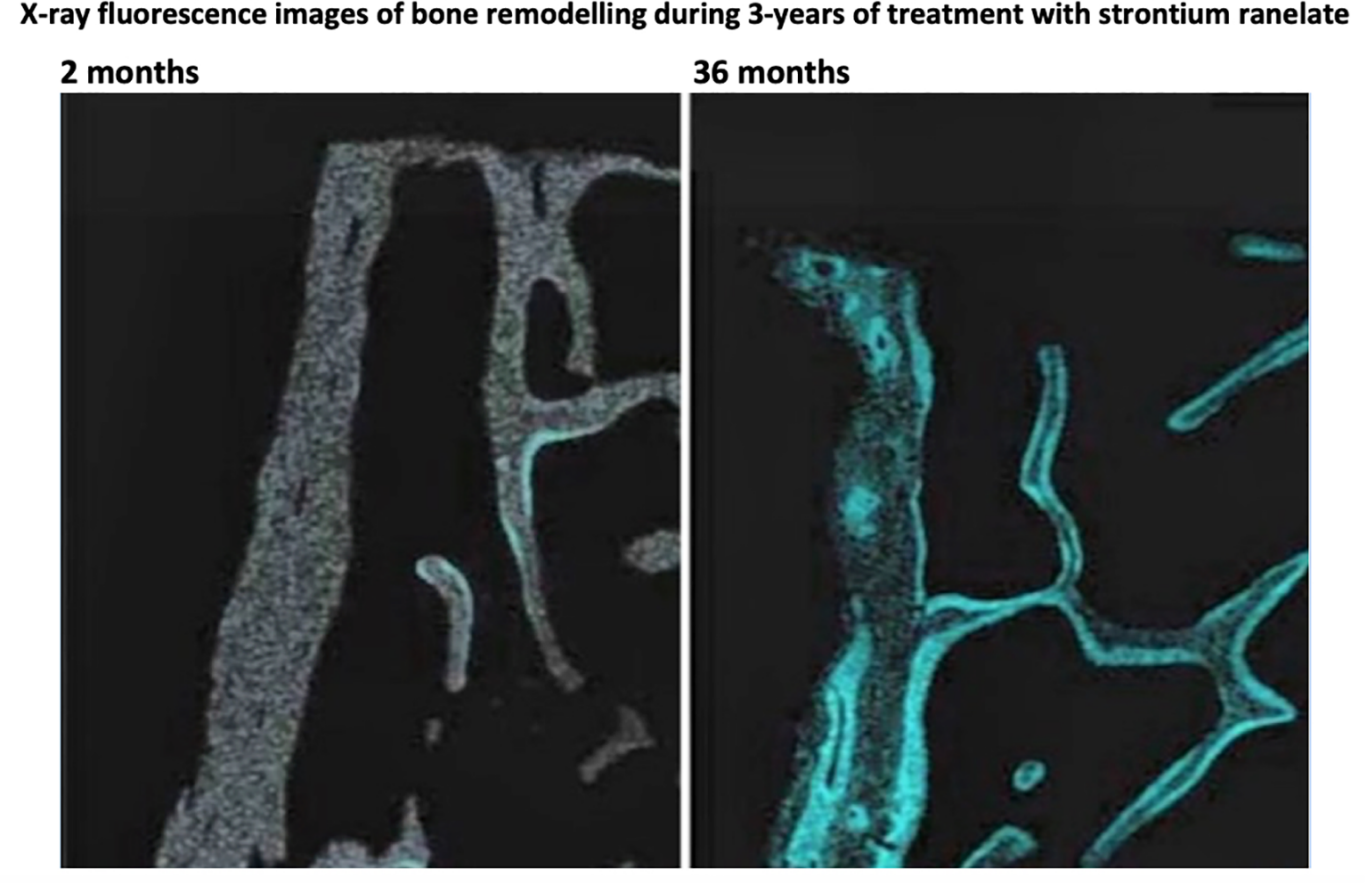
X-ray fluorescence images of bone biopsy samples illustrating the accumulation of stable strontium (in blue) at bone remodelling sites after 2 months (left) and 36 months (right) treatment for osteoporosis with daily strontium ranelate. The figure illustrates how the deposition of bone-seeking tracers in bone tissue is restricted to sites of newly mineralising bone. Adapted from Boivin et al. 2010 (10).

The PET bone scan agent [^18^F]NaF specifically targets the mineralisation phase by replacing OH^-^ ions with F^-^ ions in newly forming microcrystals of hydroxyapatite (9). Fluoride is the most electronegative ion, with a small ionic diameter and high charge density that allow it to competitively displace OH^-^. Other bone seeking radionuclides such as ^85^Sr, ^89^Sr, and ^223^Ra work instead by substituting one of the calcium ions in the hydroxyapatite molecule with an alternative alkaline earth metal. **Figure 2** shows X-ray fluorescence images of bone biopsy samples that illustrate the cumulative effects of bone remodelling based on the deposition of stable strontium during long-term daily treatment of osteoporosis with strontium ranelate (10). The figure illustrates how the deposition of bone-seeking tracers in bone tissue is restricted to sites of newly mineralising bone. For ^18^F, this hypothesis is supported by a study by Reeve et al. in patients with postmenopausal osteoporosis that reported correlations between bone blood flow measured using [^18^F]NaF, bone turnover measured using [^85^Sr]SrCl_2_, and dynamic bone histomorphometry performed at the iliac crest (11). It is also supported by more recent studies that have examined the correlations between K_i_ measurements at the spine and hip and indices of bone formation measured by bone biopsy in patients with chronic kidney disease mineral and bone disorder (CKD-MBD) (3, 4).

## 3 What is quantitative [^18^F]NaF PET-CT?

After injection into the vascular system, [^18^F]NaF is cleared from plasma into soft tissue, including the unbound bone compartment (i.e., the bone extracellular fluid [ECF]), from where the ^18^F^-^ ions compete with OH^-^ ions at sites of on-going mineralisation of bone matrix to be laid down on the surface of the newly forming microcrystals of hydroxyapatite (9). [^18^F]NaF does not metabolise in the blood; therefore, the AIF does not require a metabolite correction. [^18^F]NaF is also excreted through the kidneys by glomerular filtration followed by some degree of tubular reabsorption. Variations in the latter can be minimised by keeping the patient well hydrated to maintain a high urine flow through the kidneys (12, 13).

Quantitative [^18^F]NaF PET-CT as applied to the study of bone and mineral metabolism is a non-invasive functional imaging technique that traditionally uses a 60-min dynamic PET scan to quantify bone metabolic activity (K_i_) and regional bone blood perfusion (K_1_) rather than relying on the simple visual interpretation or semi-quantitative standardised uptake values (SUV) analysis associated with static PET scan imaging (5). PET scans are calibrated to measure voxel by voxel the regional concentration of the [^18^F]NaF tracer in units of becquerels per millilitre (Bq/mL). The CT scan is used for attenuation correction of the PET signal and is often also used for identifying the bone regions of interest (ROI). For example, the lumbar spine region can be segmented by placing elliptical ROIs on multiple transaxial CT image slices to include only the trabecular spongiosa of the vertebral bodies and exclude the cortical bone surrounding the vertebral bodies. These are subsequently transferred to the PET scan images to obtain an accurately co-registered measurement of [^18^F]NaF activity concentration.

Quantitative [^18^F]NaF PET-CT is used as an imaging biomarker that is considered to provide a measure of the site-specific rate of bone formation (3, 4, 14). However, possible confounding artefacts include atherosclerosis, vascular calcification, and inflammation. Other artefacts include degenerative processes such as osteophytes and facet joint arthritis which are marked by focal areas of increased [^18^F]NaF activity.

## 4 Why do we need quantitative [^18^F]NaF PET-CT?

X-rays, dual X-ray absorptiometry (DXA), CT scanning, and the single photon emission computed tomography (SPECT) radionuclide bone scan are the principle image-based clinical standards used in the qualitative diagnosis of musculoskeletal diseases. DXA of the spine and hip is used to quantify bone mineral density (BMD) and diagnose osteoporosis. It is also used to measure the response to anti-fracture treatment, although it often takes 2 to 3 years before any significant change in BMD can be detected in an individual patient. CT and (to a limited extent) DXA imaging provide bone structural information, while PET imaging provides information at a functional level. The response to any treatment will start at a functional level, representing molecular and cellular changes, within weeks, but can take a few months or years before it is observable at an anatomic level. Therefore, the non-invasive measurement of regional bone metabolism using quantitative [^18^F]NaF PET imaging can detect the response to the treatment of metabolic or metastatic bone diseases much earlier than DXA or CT, and therefore is an efficient way of conducting early-phase clinical trials of novel drugs to avoid later-stage attrition due to lack of efficacy, with the potential for translation to the clinic (15).

**Figure 3:**
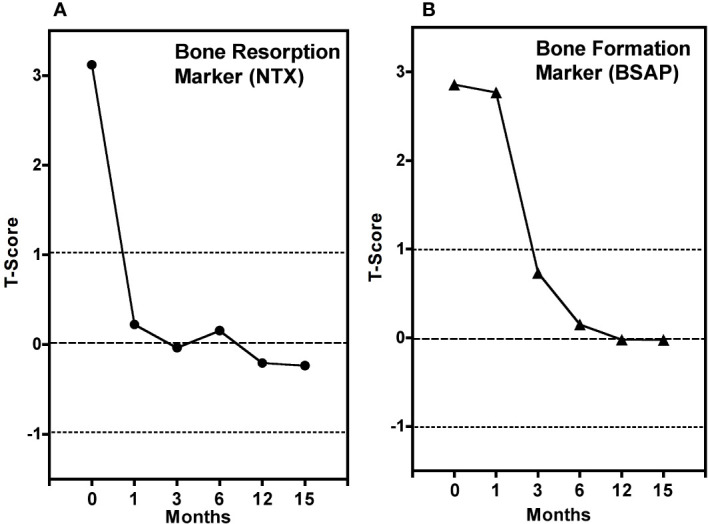
Average measurements of biochemical markers of **(A)** bone resorption (NTX) and **(B)** bone formation (BSAP) **(B)** at six time points in 85 postmenopausal women starting alendronic acid treatment for postmenopausal osteoporosis. Bone marker measurements are expressed in T-score units, in analogy with bone mineral density T-scores, relative to a group of 46 non-osteoporotic premenopausal women, without treatment, for whom T-score = 0. Adapted from Garnero et al. 1994 (16).

Biochemical markers of bone formation or bone resorption in serum or urine also provide an early, non-invasive measurement of bone metabolism, which can be used to verify response within a few weeks after the commencement of treatment (16, 17). Commonly used bone resorption markers include serum carboxy-terminal collagen crosslinks (CTX, also known as C-terminal telopeptide) and N-terminal telopeptide (NTX, also known as amino-terminal collagen crosslinks). Commonly used bone formation markers include serum bone-specific alkaline phosphatase (BSAP) and serum procollagen 1 N-terminal propeptide (P1NP). **Figure 3** shows the changes in biochemical markers of bone resorption and bone formation in a large study of patients starting alendronic acid treatment for postmenopausal osteoporosis (16). Treatment with a potent anti-resorptive agent leads to a rapid decrease in the bone resorption markers NTX within 1 month. However, due to the remodelling cycle (**Figure 1**), the subsequent decrease in the bone formation marker BSAP was not seen until 3 to 6 months after the start of treatment. Biochemical markers provide a rapid and simple way of measuring whole-body skeletal metabolism. However, unlike imaging methods such as [^18^F]NaF PET-CT, they are unable to provide site-specific information at clinically important sites susceptible to fractures, such as the hip and spine.

**Figure 4:**
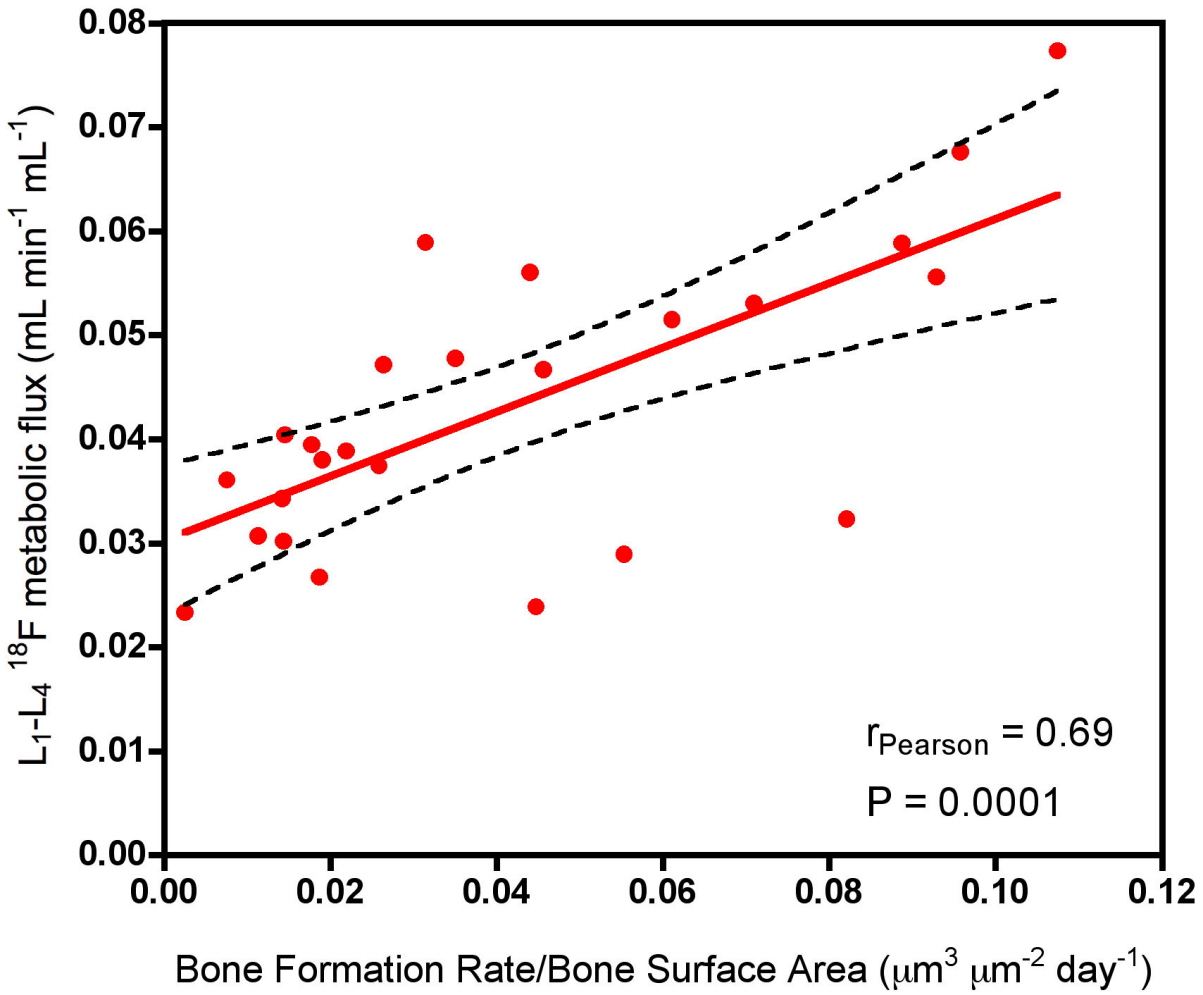
Scatter plot showing the correlation between K_i_ values at the lumbar spine measured using [^18^F]NaF PET and the bone formation rate per unit bone surface area measured at the iliac crest using bone biopsy with tetracycline labelling. Adapted from Aaltonen et al. 2020 (3).

The imaging technique of quantitative [^18^F]NaF PET-CT can provide information about changes in regional bone formation within about 12 weeks after the commencement of treatment, much earlier than the changes in BMD measured by DXA (15). The link between quantitative measurements of [^18^F]NaF kinetics and bone formation rate is supported by a study by Reeve et al. that used both [^18^F]NaF and [^85^Sr]SrCl_2_ together with dynamic bone histomorphometry to establish correlations between skeletal blood flow, calcium-surrogate bone mineral uptake, and mineral apposition rate in patients with postmenopausal osteoporosis (11). In more recent studies, bone metabolic flux (K_i_) values obtained using [^18^F]NaF PET have been compared with measurements of bone formation rate made by the gold standard method of bone biopsy in patients with CKD-MBD (**Figure 4**) (3, 4). By comparison with [^18^F]NaF PET-CT, bone biopsy is a painful and complex procedure that is limited to a single biopsy site at the iliac crest, is subject to significant measurement errors and is now rarely performed, with many centres not having the necessary resources.

## 5 What can we estimate from quantitative [^18^F]NaF PET-CT?

There are two main approaches to the quantitative measurement of bone metabolism using [^18^F]NaF PET-CT, namely the measurement of SUV and K_i_, respectively.

A SUV measurement provides a semi-quantitative measure of tracer uptake within a defined volume of interest (VOI) at a specific time after injection and is calculated by normalising the [^18^F]NaF concentration in the bone VOI measured in units of Bq/mL for the injected activity (Bq) and the patient’s body weight (g), and expressed in units of g/mL. For diffuse metabolic bone diseases such as osteoporosis, SUV is usually calculated from the mean tracer concentration within the VOI (SUV_mean_). The alternative SUV_max_ and SUV_peak_ values are often used in oncology for studies using [^18^F]FDG, where clinicians tend to follow a clear framework and guidelines for their use (18). However, there is still no agreed protocol on how to define SUV_peak_ values, and this can have an impact on the measurement of response to treatment (18). Further work is needed to assess if SUV_max_ or SUV_peak_ can add value to bone imaging using [^18^F]NaF PET and to establish the optimum way of measuring and reporting these parameters to minimise methodology-related variability (18, 19).

The measurement of SUV is popular due to its simplicity and relatively small precision error (i.e., reproducibility error) of around 10%, compared to the greater complexity of measuring K_i_ values, with somewhat larger precision errors of typically 11-15% (20). Good reproducibility requires the standardisation of both clinical and scanner protocols, as well as regular quality assurance (QA). Various initiatives have recently been proposed for the harmonisation of PET scanners that may be beneficial to minimise calibration and reproducibility-related errors (21–23). Another potential disadvantage of SUV measurements is that they are not independent of the [^18^F]NaF kinetics at other sites in the body (24). SUV measurements are affected by competition from the kidneys and bone lesions in other parts of the skeleton for the finite amount of bone tracer given at injection, and this competition may differ substantially from patient to patient due to variations in the whole-body metabolism of the tracer. Consequently, SUV has been shown to be less accurate for measuring response to treatment than K_i_ in some circumstances, for example treatment with a drug that has a strong effect on the bone remodelling rate (24), in patients with extensive metastatic bone disease, or patients with extensive Paget’s disease.

**Figure 5:**
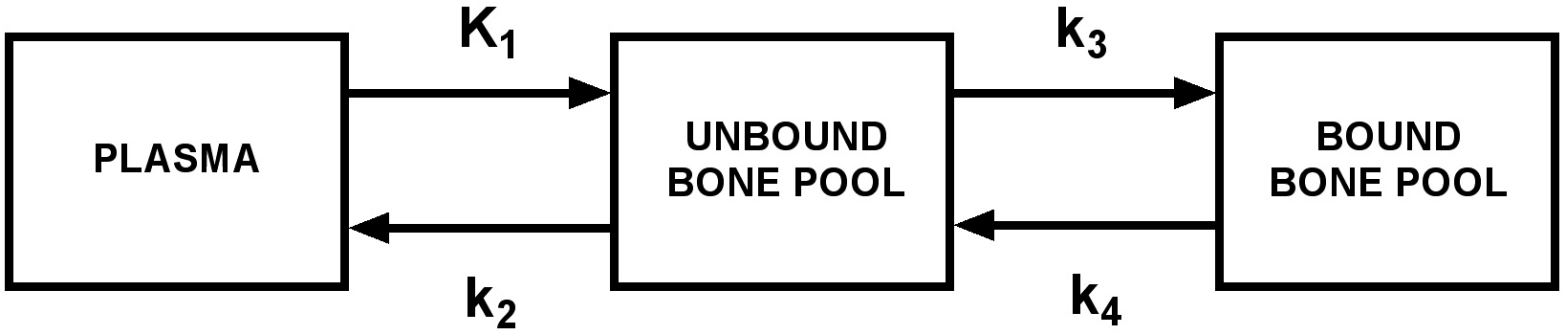
The Hawkins compartmental model describing the kinetics of [^18^F]NaF in bone. Bone metabolic flux K_i_ representing the net transfer of [^18^F]NaF from plasma to bone mineral is calculated from the equation: $K_{i}=K_{1}\times \frac{k_{3}}{\left(k_{2}+k_{3}\right)}$ .


Traditionally, the measurement of K_i_ values has required a 60-minute dynamic scan in a single bed position to measure the bone time-activity curve in the chosen VOI, together with information on the arterial input function obtained either by direct arterial sampling or from a VOI placed in the left ventricle or the aorta. The two time activity curves are solved together to find the rate constants in the Hawkins compartmental model, which consists of a bone extracellular fluid (ECF) compartment and a bound bone compartment that are supplied with [^18^F]NaF tracer from plasma (**Figure 5**) (5). The rate constant K_1_ (units: mL/min/mL) measures the amount of tracer being cleared each minute from plasma into the ECF compartment in each mL of the bone VOI. Because ^18^F^-^ in solution readily forms molecules of neutral hydrogen fluoride, it is highly diffusible, and at the blood flow rates found in bone, K_1_ approximates to a measurement of bone blood flow (25). Once the tracer is in the bone ECF compartment, some of it is laid down in bone mineral with a rate constant k_3_ (units: min^-1^), while the rest diffuses back into the vascular system with a rate constant k_2_ (units: min^-1^). There is also a small efflux of the tracer in bone mineral back into the ECF compartment with a rate constant k_4_ (units: min^-1^). The rate constant k_4_ is small and is often ignored. Once the values of K_1_, k_2_, and k_3_ are known by solving the compartmental model, the value of K_i_ (units: mL/min/mL) is calculated from the net rate of transfer of tracer from the vascular system to the bound bone compartment:

(1) $$K_{i}=K_{1}\times \frac{k_{3}}{\left(k_{2}+k_{3}\right)}$$

Measured values of K_i_ in the lumbar spine are typically around 0.03 mL/min/mL, which means that the net quantity of [^18^F]NaF tracer laid down in bone in each mL of the bone VOI in one minute is equal to the amount contained in 0.03 mL of plasma.

Unlike SUV, K_i_ values are considered fully site-specific because they measure the local metabolic flux in the chosen VOI with respect to the concentration of tracer in the artery supplying the site under investigation. Thus, K_i_ values remain unaltered by the tracer uptake occurring at other sites in the body not under investigation. Therefore, in cases where the bone disease or its treatment is potent enough to alter the area under the [^18^F]NaF arterial input function curve due to aggressive uptake of tracer in other parts of the body, there is a decoupling of the usually strong correlation between SUV and K_i_, and the latter is the more site-specific measurement (24). Otherwise, the two measurements SUV and K_i_ remain well correlated. This is the primary reason why in some circumstances the changes observed after bone treatment by measuring K_i_ values may differ from those observed by SUV.

The difference in the results between SUV and K_i_ measurements can have important implications for the design of clinical trials. The number of subjects required to demonstrate a statistically significant response to a drug treatment depends on the ratio of the treatment effect of the chosen parameter (i.e., SUV or K_i_) to the precision error. The treatment effect, measured as a percentage change between the baseline and follow-up scans, depends on the sensitivity of the parameter used to make the measurements. A parameter that has a larger value of the ratio of treatment effect to precision error will be more cost-effective in research studies because fewer subjects will be required to achieve a statistically significant result (20). The precision errors at our centre for SUV and K_i_ are around 10% and 11-15% (20) respectively, but the capability of K_i_ to measure treatment effect (i.e., the sensitivity) can in some circumstances be substantially higher than SUV (20, 24).

## 6 Is there an easier way to measure K_i_ values using [^18^F]NaF PET-CT?

The requirement to solve the Hawkins compartmental model to find the rate constants K_1_, k_2_ and k_3_ before K_i_ can be calculated can be avoided by using the Patlak plot graphical method (26). This is especially attractive if it is assumed that the rate constant k_4_ is negligible, although in practice with this assumption the values of K_i_ obtained are slightly reduced. In the Patlak plot method, the bone time activity curve and the arterial input function are plotted so the points can be fitted with a straight line whose slope gives the value of K_i_ (26):


(2) $$\frac{C_{bone}\left(T\right)}{C_{plasma}\left(T\right)}=K_{i}\times \frac{\int_{0}^{T}C_{plasma}\left(t\right)dt}{C_{plasma}\left(T\right)}+V_{o}$$

**Figure 6:**
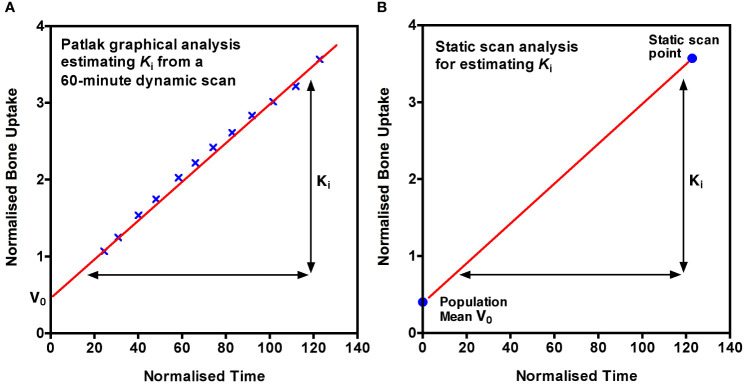
**(A)** The Patlak plot graphical method of calculating K_i_. Normalised bone uptake is plotted against normalised time from 10 to 60 minutes after injection of tracer and the linear regression line fitted. The slope of the line is K_i_ and the intercept is the volume of distribution of the tracer in the bone extracellular fluid (ECF) compartment, V_0_. The mathematical definitions of normalised uptake and normalised time are given in the text. **(B)** The static scan method of measuring K_i_ is a simplified form of the Patlak plot with a single scan point at about 60 min after injection with the intercept replaced by the population mean value of V_o_.

In Equation 2, C_bone_(T) is the concentration of [^18^F]NaF in the bone VOI (units: Bq mL^-1^) at time T after tracer injection, C_plasma_(T) is the concentration in plasma (units: Bq mL^-1^) at time T, V_o_ is the volume of distribution of tracer in the bone ECF (i.e., the fraction of the total bone VOI occupied by the ECF compartment), and $\int_{0}^{T}C_{plasma}\left(t\right)dt$ is the area under the plasma AIF curve over the time interval between 0 and T. Equation 2 is a linear equation of the form Y = m*X + c, where Y = $\frac{C_{bone}\left(T\right)}{C_{plasma}\left(T\right)}$ is referred to as the normalised bone uptake and X = $\frac{\int_{0}^{T}C_{plasma}\left(t\right)dt}{C_{plasma}\left(T\right)}$ is referred to as the normalised time (**Figure 6A**). The data plotted on the Y- and X-axes between 10–60 min (the non-linear part during the early non-equilibrium state of the system between 0-10 min is ignored) is fitted by linear regression to obtain the values of K_i_ and V_o_. It is important to note that, unlike the full solution of the Hawkins model, the Patlak plot method only provides measurements of bone metabolic flux (K_i_), not values of bone blood flow (K_1_).

We have recently described a simplified imaging protocol in which the Patlak plot shown in **Figure 6A** is reduced to two points, one obtained from a single short (3 to 4 minute) static PET-CT scan acquired at around 60 minutes after tracer injection and the second from the population mean value of V_o_ (**Figure 6B**) (26). The AIF data used to calculate the X and Y values at the single static scan point in **Figure 6B** is obtained from 2 or 3 venous blood samples taken between 30 and 60 minutes after injection which are used to define the terminal exponential of the AIF (7). A population average residual function representing the peak of the bolus injection and the early fast exponential components is added to the terminal exponential to approximate the full 0 to 60 min AIF (7). In practice, around 75 to 80% of the area under the plasma clearance curve between 0 and 60 min comes from the terminal exponential, thus limiting the effects of patient-to-patient variations in the bolus peak on the values of K_i_. An important advantage of the static scan technique is that a series of short static scans can be acquired at several bed positions, enabling K_i_ values to be measured at multiple sites with only a single injection of tracer. A major aim of the method is to achieve the best possible accuracy and precision (i.e., the lowest reproducibility errors) in order to minimise the number of participants required in research studies. We hope that this method will make the technique more accessible to researchers and clinicians and promote the use of quantitative [^18^F]NaF PET imaging in the clinic as well as in research settings (6).

## 7 Clinical applications, future developments, and limitations

[^18^F]NaF PET-CT scans have been used to study a variety of metabolic, metastatic, and other bone diseases, often with the aim of quantifying response to treatment and investigating the efficacy of bone-directed therapies (**Table 1**). A recent book edited by Kairemo and Macapinlac provides a detailed account of clinical applications (36). An explanation of cellular mechanisms and the use of [^18^F]NaF PET-CT as a diagnostic modality for metabolic, autoimmune, and osteogenic bone disorders has been published by Park et al. (37).

**Table 1:** Key examples of clinical applications of [^18^F]NaF PET-CT.

Medical Condition	Specific Disease (with references)	Patient Type	Sample Size	Major Findings
Metabolic bone disorders	Osteoporosis (15)	Postmenopausal women	27	[^18^F]NaF PET an imaging biomarker for treatment efficacy at the hip
	Paget’s disease (27)	Vertebral Paget’s disease	14	Measurement of [^18^F]NaF kinetic parameters in Paget’s disease
	Chronic kidney disease mineral and bone disorder (28)	Renal dialysis patients	26	Investigation of [^18^F]NaF PET as a tool for investigating adynamic bone disease in CKD-MBD patients
	Medication-related osteonecrosis of the jaw (29)	Multiple myeloma patients	26	SUV elevated in patients with bisphosphonate related ONJ
Metastatic bone disease	Breast cancer (30)	Breast cancer patients	12	K_i_ measurements in bone metastases identified patients with clinically progressive disease more reliably than SUV
Atherosclerosis	Coronary atherosclerosis (31)	Patients with myocardial infarction and stable angina	92	[^18^F]NaF identified patients with ruptured and high-risk coronary plaques
	Aortic syndrome (32)	Patients with acute aortic syndrome	67	[^18^F]NaF PET/CT holds promise as a marker of disease severity in patients with acute aortic syndrome
Autoimmune diseases	Rheumatoid arthritis (33)	Adult patients with rheumatoid arthritis	17	Joint uptake measured using [^18^F]NaF PET/CT accurately predicted disease activity
Osteogenic bone disorder	Fibrodysplasia ossificans progressiva (FOP) (34)	Patients with possible progression of heterotropic ossification	5	[^18^F]NaF PET/CT identified patients with asymptomatic but progressive heterotopic ossification lesions
Sports medicine	Assessment of acute bone loading (35)	Healthy subjects	12	Bone loading induced an acute response in Hawkins model parameters and SUV

One application that has attracted interest is the potential of [^18^F]NaF PET-CT scans to help with the investigation of patients with CKD-MBD, in particular whether measurements of lumbar spine K_i_ can differentiate patients with high turnover disease such as secondary hyperparathyroidism from those with low bone turnover such as adynamic bone disease (ABD) (3, 4, 38, 39) and improve on the sensitivity and specificity of existing diagnostic methods based on measurements of intact parathyroid hormone (iPTH) and other bone turnover markers (40). An interesting aspect of these studies is that, in addition to providing data on potential diagnostic thresholds for K_i_ for the detection of ABD, they also involve measurements of bone turnover using bone biopsy with tetracycline labelling at the iliac crest. A limitation is that these studies are single-centre with no more than 20 to 30 participants each. Although the resulting scatter plots of K_i_ against histomorphometric measurements of bone formation from the bone biopsy are inevitably noisy, there is a consistent trend toward finding positive relationships that confirm the basic tenet that quantitative [^18^F]NaF PET-CT provides a measurement related to bone formation rate.

Further progress in this field will require the establishment of a multi-centre study that includes many more CKD-MBD cases and the adoption of a common protocol for scan acquisition and analysis. An international imaging consortium could play a role in the discovery, validation, and delivery of innovative, quality-assured imaging biomarkers for clinical use. Such a consortium would allow data from multiple centres to be pooled and analysed together and would further build on agreed-upon standardised quality assurance protocols. This may help design new multi-centre trials using the chosen [^18^F]NaF PET-CT protocol.

The published literature already includes many small single-centre studies, most of them with baseline [^18^F]NaF PET-CT scans in untreated participants, that could provide a potential resource to better define diagnostic ranges of K_i_ values in different disease types. Retrospective reanalysis of these scans with a common agreed-upon protocol could provide a large pool of data to help reach clinically significant conclusions and define diagnostic ranges of K_i_ values associated with abnormally low and high turnover compared to healthy bone turnover rates. The diagnostic range of K_i_ values may require adjustment for factors such as age, sex, race, disease status, treatment, PET**-**CT scanner model, and the imaging protocol.

Therefore, we believe that efforts should be made towards building a consortium that will pool resources from multiple centres to develop a database to securely store, share, and analyse previously acquired dynamic [^18^F]NaF PET-CT datasets. A database of pooled studies from worldwide centres will provide us with the ability to answer clinical questions with higher statistical power than is currently possible. It will provide a facility to develop and train tools in the area of artificial intelligence and machine learning to develop newer PET image processing, analysis, and reconstruction methods (41) to accurately estimate image-derived input functions from a 60-min dynamic PET scan so that the need to obtain blood samples can be avoided in the future (42). When combined with automated methods of performing bone image segmentation to generate time-activity curves, these inputs could be used to calculate K_i_ values automatically at multiple skeletal sites and help increase the throughput in the clinic and research settings. With the emergence of total body PET systems, kinetic and static quantitative measurements of skeletal activity will likely become faster and even more accurate (28, 43–46). Furthermore, total-body PET scanners will enable K_i_ values to be estimated from static PET scans (3, 38) as well as enable PET data to be visualised as parametric (K_i_ or SUV) images with a calibrated color scale to make it easier for the clinical end-users to interpret the findings. A combination of total-body PET scanners and artificial intelligence may help revolutionise the entire medical imaging domain in the future (47).

There are several limitations associated with [^18^F]NaF PET-CT imaging that should be noted. PET scanners have a spatial resolution of around 6 mm, which is inferior to CT and MRI imaging. While measurements at the spine and hip are unaffected by the partial volume effect, reliable measurements of narrower bones such as the radius require a correction for the spill-over of counts outside the bone volume imaged by CT. If necessary, a scanner-specific calibration curve can be obtained by imaging a phantom with cylindrical rods of varying diameters, and an appropriate correction factor can be derived. The dependence of image-derived arterial input functions on the reconstruction algorithm could, unless controlled, be a limitation for harmonising data from different studies and can significantly affect K_1_ as well as K_i_ values (48). The radiation dose to the patient is also a significant consideration and can be minimised by using an injected activity as low as 90 MBq, which reduces the effective dose from the [^18^F]NaF tracer to 1.5 mSv, compared with the activity of 250 MBq (4.3 mSv) recommended for clinical imaging (49). There is also a small radiation dose from the low-dose CT acquisition required for attenuation correction (and anatomical mapping in some cases), but this is dependent on the exact scan fields chosen.

## 8 Conclusion

Bone metabolic flux (K_i_) values obtained using quantitative [^18^F] sodium fluoride PET-CT imaging provide a measurement indicative of the local rate of bone formation. K_i_ is more accurate than standardised uptake values (SUV) for measuring response to treatment when studying drugs that have a potent effect on bone remodelling across the whole skeleton. K_i_ can be estimated at multiple sites in the skeleton without loss of accuracy or precision with a single injection of tracer using a simplified procedure involving a series of 4-minute static PET scans, 2-3 venous blood samples, aided by an Excel spreadsheet to perform the calculations (6). These latter may help avoid the need for direct arterial sampling in similar studies in the future and simplify the application of the [^18^F]NaF PET-CT technique, allowing its wider spread in the community.

## Author contributions

All authors contributed to the preparation of the manuscript. The first draft of the manuscript was written by TP and all authors commented on previous versions of the manuscript. GB is the senior/last author. All authors contributed to the article and approved the submitted version.
